# Madelung and Hubbard interactions in polaron band model of doped organic semiconductors

**DOI:** 10.1038/ncomms11948

**Published:** 2016-09-01

**Authors:** Rui-Qi Png, Mervin C.Y. Ang, Meng-How Teo, Kim-Kian Choo, Cindy Guanyu Tang, Dagmawi Belaineh, Lay-Lay Chua, Peter K.H. Ho

**Affiliations:** 1 Department of Physics, National University of Singapore, Lower Kent Ridge Road, Singapore S117550, Singapore; 2 Solar Energy Research Institute of Singapore (SERIS), National University of Singapore, 7 Engineering Drive 1, Singapore S117574, Singapore; 3 Department of Chemistry, National University of Singapore, Lower Kent Ridge Road, Singapore S117552, Singapore

## Abstract

The standard polaron band model of doped organic semiconductors predicts that density-of-states shift into the *π*–π* gap to give a partially filled polaron band that pins the Fermi level. This picture neglects both Madelung and Hubbard interactions. Here we show using ultrahigh workfunction hole-doped model triarylamine–fluorene copolymers that Hubbard interaction strongly splits the singly-occupied molecular orbital from its empty counterpart, while Madelung (Coulomb) interactions with counter-anions and other carriers markedly shift energies of the frontier orbitals. These interactions lower the singly-occupied molecular orbital band below the valence band edge and give rise to an empty low-lying counterpart band. The Fermi level, and hence workfunction, is determined by conjunction of the bottom edge of this empty band and the top edge of the valence band. Calculations are consistent with the observed Fermi-level downshift with counter-anion size and the observed dependence of workfunction on doping level in the strongly doped regime.

## Abstract (web summary)

Conducting polymers with high workfunction are desirable for making ohmic contacts at electrode/organic semiconductor interfaces for electronic applications. Png *et al*. show workfunction up to 6 eV in hole-doped triarylamine-fluorene copolymers, explained by including electron correlation effect.

## Graphical abstract

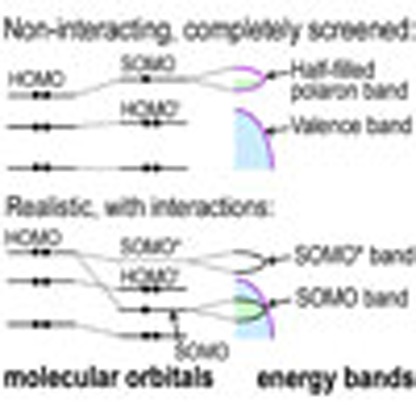

High workfunction electrodes are needed to make ohmic hole contacts to organic and other semiconductor layers for efficient device operation^1^. Hole-doped conducting polymers are of particular academic and technological interest because of their ability to make ohmic hole contacts to a variety of semiconductors for light-emitting diode, photodiode, solar cell and transistor applications. However, practical hole-doped conducting polymers with good stability, adequate processability and reliable performance have so far been limited primarily to the thiophene families, such as poly(3,4-ethylenedioxythiophene): poly(styrenesulfonic acid)^2^ and sulfonated poly[3-(2-(2-methoxyethoxy)ethoxy)thiophene-2,5-diyl]: poly(4-hydroxystyrene) (S-P3MEET:PHOST)^3^. Although the vacuum workfunctions *ϕ* of these polymers are high, ≈5.2 eV, they are still not high enough to furnish ohmic hole contacts to organic semiconductors (OSCs) with ionization potentials *I*_p_ ≳5.4 eV, many of which are of great technological interest^4^,^5^. Although the insertion of an insulating dipolar surface layer with the correct polarity can tune *ϕ*^6^,^7^, the attendant increase in kinetic barrier for charge injection ultimately limits its usefulness^8^,^9^.

In the course of our work to find a new *π*-conjugated core that can provide a larger inherent *ϕ*, that is, without relying on surface dipolar layers, we developed a family of hole-doped triarylamine−fluorene (TAF) copolymers (Fig. 1a). The *ϕ* values that can now be attained in some of these doped polymers are even larger than their pristine (undoped) *I*_p_. The density-of-states (DOS) recedes from the *π*–π* gap upon hole doping, in apparent contradiction to prevailing literature expectation^10^,^11^,^12^,^13^. This compels us to revisit the standard polaron band theory^10^,^11^,^12^,^13^. The present Letter reports both development of these ultrahigh workfunction hole-doped TAF copolymers, and revision of the standard polaron band theory by incorporating Hubbard and Madelung interactions to achieve agreement with experiment. This revision fundamentally changes the understanding of polaron band energetics. The result produces correct predictions for workfunction, a central property of doped OSCs important for much of device physics. Although the need to incorporate such Coulomb interactions is compelled by analysis of the hole-doped TAF polymers, it is general and applies to doped OSCs wherever electron correlation effects cannot be neglected.

The triarylamine–triarylaminium system has been relatively little explored as potential hole-injection layers (HILs)^14^,^15^, despite the known stability of triarylaminium cations^16^ and the high electrical conductivity of their polymers (10^−2^–10^0^ S cm^−1^)^17^,^18^. These polymers have been investigated some time ago for electrochromism and high-spin ferromagnetism^19^. Undoped triarylamine oligomers and polymers on the other hand are widely used today as hole-transport layers for OSC diodes^20^,^21^. We show here that when hole doped, they can in fact exhibit ultrahigh inherent workfunction to give more efficient HILs than previously known. HILs are different in their function and requirements from hole-transport layers. HILs have to be heavily p-doped to a sufficiently high carrier density to make ohmic contact with the adjacent metallic electrode. They also need to exhibit a sufficiently high and well-defined workfunction to make ohmic contact to the adjacent semiconductor in the device.

## Results

### TAF copolymers

The solubility challenge of triarylaminium polymers^17^,^18^ has been resolved here by copolymerizing the desired triarylamine units with 9,9-dioctylfluorene-2,7-diyl units to give the TAF copolymers. Systematic substitution of the pendant ring of the triarylamine unit affords a tunable *I*_p_ over a one electronvolt wide range. The following TAF copolymers (weight-average molecular weight, 100–200 kDa) were investigated: poly(9,9-dioctylfluorene-2,7-diyl-1,4-phenylene-*N*-(*p*-sec-butylphenyl)imino-1,4-phenylene) TFB (that is, *p*-*sec*-butyl substituted; *I*_p_, 5.5 eV), mTFF (*m*-trifluoromethyl; 5.9 eV), pTFF (*p*-trifluoromethyl; 6.25 eV) and TFBF (2,5-bis(trifluoromethyl) disubstituted; 6.3 eV). *I*_p_ values were obtained from extrapolation of the valence band (VB) onset measured by ultraviolet photoemission spectroscopy (UPS). The substitution also advantageously suppresses undesirable dimerization reactions of the triarylaminium cations^16^. This TAF family provides an excellent set of model OSCs to examine the effect of doping on electronic structure. They exhibit non-dispersive and hence well-resolved frontier *π*-electron bands. Furthermore, their partially doped states are important prototypes of the weak-to-intermediate coupled mixed valence materials of great current interest^22^,^23^.

### Hole-doped TAF copolymers

**Figure 1:**
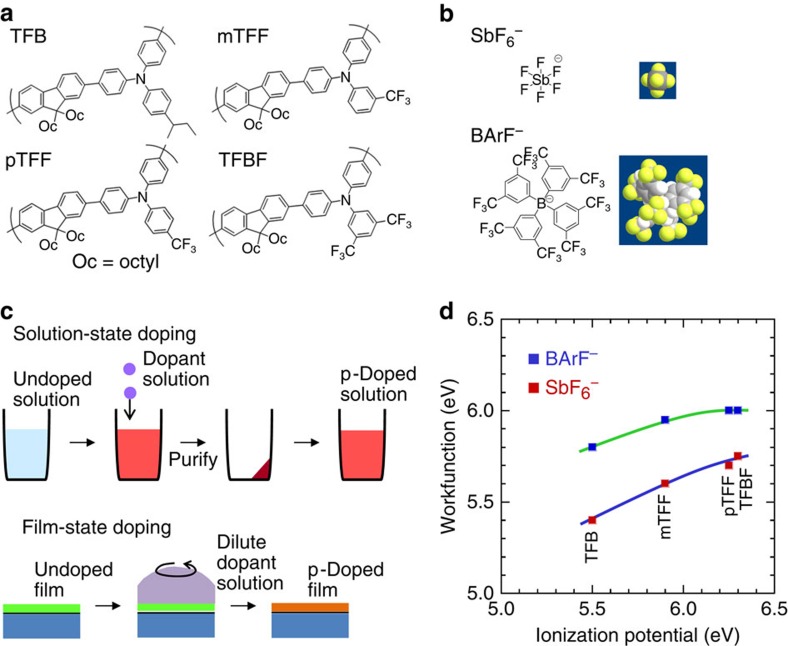
Chemical structures and p-doping methodologies. (**a**) Chemical structures of TAF copolymers. (**b**) Chemical structures of counter-anions. van der Waals surface: F=yellow, H=white, C=light grey, Sb=dark grey. Effective van der Waals diameters: SbF_6_^−^, 6.7 Å´; BArF^−^, 16 Å´. (**c**) Schematic of solution-state and film-state doping methodologies. (**d**) UPS workfunctions of strongly hole-doped films with SbF_6_^−^ or BArF^−^ as counter-anion at DL≈0.5−0.9 *h*^+^/r.u., plotted as a function of the solid-state ionization potentials of the undoped films.

Hole-doped TAF copolymers with the desired doping level (DL) were generated using nitrosonium hexafluoroantimonate (NOSbF_6_) oxidation. NO^+^ is a strong one-electron oxidant that has been successfully used to hole-dope polymer OSCs with high *I*_p_ of up to 5.9 eV^24^. This process can be carried out in solution (Fig. 1c, see also Methods: p-Doping) with a doping efficiency of near unity, much higher than the weaker molecular dopants^25^. The doped polymers were purified by precipitation–redissolution cycles and then spin-cast or printed into films. The mobile holes on the TAF backbone are counter-balanced by SbF_6_^−^ or tetrakis(3,5-bis(trifluoromethyl)phenyl)borate (BArF^−^; Fig. 1b) through ion exchange. The van der Waals diameter of BArF^−^ is about two and a half times that of SbF_6_^−^. Both anions are chemically unreactive and have molecular orbitals well outside the *π*–π* gap of the polymers. They provide simple charge compensation with no intermolecular hybridization with the *π* and π* bands of the polymer^26^.

Alternatively, hole doping can be performed by contacting photo-crosslinked^27^ TAF copolymer films (Fig. 1c) with a dilute solution of the dopant on a spinner in the N_2_ glovebox (*p*O_2_, *p*H_2_O<1 p.p.m.). The crosslink density used (6.5 mol/mol%) is sufficient to prevent film dissolution, but does not degrade opto(electronic) and other semiconductor properties. Hole doping leads to the expected emergence of *P*_1_ and *P*_2_ subgap polaron absorptions at 1.0 and 2.5 eV respectively (see Supplementary Fig. 1), similar to molecular triarylaminium salts^19^. This indicates the holes are singly-charged polarons residing at the N sites, up to full doping of 1 hole per repeat unit (*h*^+^/r.u.) (vide infra). The polaron absorption band intensity also scales linearly with loss of the π*←*π* absorption band intensity^19^. This indicates the polarons are well-behaved and relatively decoupled with weak intra- and inter-chain interactions.

Figure 1d plots the *ϕ* of hole-doped TAF:SbF_6_ and TAF:BArF films against their pristine *I*_p_ values. These *ϕ* values were obtained in the usual way from the kinetic energy of photoelectrons emitted from the Fermi level (FL), established by a sputtered-clean Ag reference (*E*_k, FL_) and the low-energy cutoff (*E*_k, LECO_) of the sample: *ϕ*=*E*_k, LECO_+h*ν*−*E*_k, FL_, where *hν* is the photon energy (He I, 21.21 eV). The *ϕ* of heavily doped films (0.5≲DL≲1 *h*^+^/r.u.) increases with *I*_p_, from 5.4 eV for TFB:SbF_6_ to 5.75 eV for pTFF:SbF_6_ and TFBF:SbF_6_ (Fig. 1d). These values exceed the 5.3-eV threshold for oxidation of the H_2_O/O_2_ couple in pH neutral water^28^. Thus, we denote them as ‘ultrahigh' workfunctions. Nevertheless, their p-doped solutions are stable for months in anhydrous acetonitrile, nitromethane and propylene carbonate when kept in N_2_, and their thin films are stable for hours in the ambient (22 °C; relative humidity 65%). This provides an advantage over the ultrahigh workfunction metal oxides, which need to be processed in vacuum^29^. When SbF_6_^−^ is exchanged for BArF^−^, even higher *ϕ* of up to 6.0 eV has been attained for pTFF:BArF and TFBF:BArF (Fig. 1d). Moreover, the *ϕ* of TFB:BArF and mTFF:BArF clearly exceed their pristine *I*_p_, which is not anticipated.

### Standard polaron band model

In the standard polaron band model^10^,^11^,^12^,^13^, the hole polaron level in an isolated molecule is taken to be the highest-occupied molecular orbital (HOMO) of the neutral state in the distorted molecular geometry of the polaron. Therefore the energy of this level relative to vacuum level (VL) is the *I*_p_ of the ‘distorted' ground state, which has to be smaller than both the vertical *I*_p_ and adiabatic *I*_p_ of the ground state. As a consequence, the hole polaron level lies inside the *π*–π* gap of the ground state. Then this distorted HOMO is assumed to be the singly-occupied molecular orbital (SOMO) of the polaron state. Broadening by intermolecular interactions produces a partially-filled polaron band. This gives rise to the widely accepted notion that hole (and electron) doping shifts valence (conduction) DOS into the gap to give *ϕ*<*I*_p_ (*ϕ*>electron affinity for electron doping). Although some experiments have been interpreted to support this notion^30^,^31^, several puzzling features persisted, including the absence of a perceptible FL step and, in some cases, the lack of any shift.

This model assumes a vanishing Hubbard gap, that is, no electron–electron interaction in the SOMO, and perfect screening of the frontier orbitals, that is, no Coulomb charging or Madelung potential interactions with counter-ions and other carriers. Both these assumptions fail for OSCs where strong electron correlation dominates. Although the standard model has provided an energy level diagram that correctly predicts the *P*_1_ and *P*_2_ optical excitations within the polaron, it must break down when energy levels are benchmarked against an external reference. Theoretical work has long pointed to a substantial Hubbard gap in OSCs^32^, while recent experimental work has uncovered a large spectator-ion effect that indicates non-negligible Coulomb interactions^33^. A study of C_60_ at metal interfaces has recently also suggested a breakdown of the standard polaron model due to Hubbard interaction^34^. However the experiments here provide the first clear evidence for large on-site and off-site Coulomb interactions, that is, Hubbard and Madelung interactions, determining *ϕ*. This has led here to *ϕ*>*I*_p_, which compels a revision of the standard polaron band model to properly treat bulk carrier doping. We show that the revised model incorporating these interactions produces new features in orbital and band ordering that may appear counter-intuitive, but agree with experiments.

### Doping-induced band structure evolution

**Figure 2:**
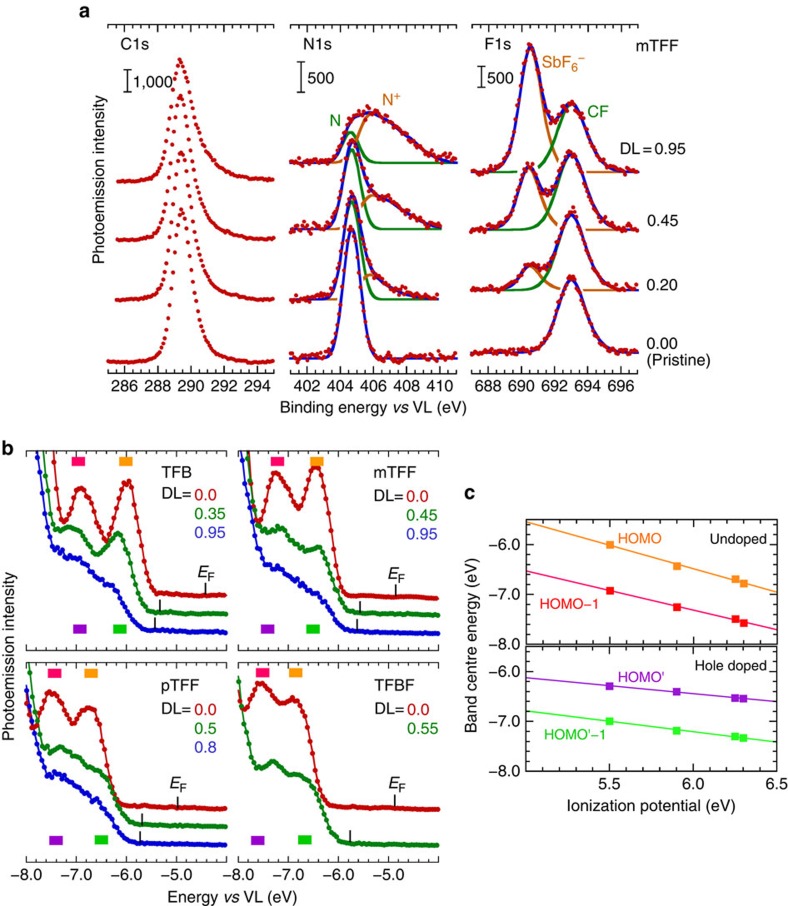
Evolution of chemical states and workfunction of TAF:SbF_6_ with DL. (**a**) C1s, N1s and F1s XPS core-level spectra plotted against BE measured from the VL as a function of DL given in hole per repeat unit (*h*^+^/r.u.) to the nearest 0.05 *h*^+^/r.u. The spectra were self-consistently fitted with spectral functions each comprising a constraint sum-of-Gaussians to simulate the undoped and doped states. (**b**) Film-state ultraviolet photoemission VB spectra plotted against energy measured from VL as a function of DL. The Fermi energies (*E*_F_) are marked. The DFT computed band positions are shown as coloured bars after a rigid energy shift to accommodate solid-state effects. undoped film: orange=HOMO, magenta=HOMO-1; doped film (1 *h*^+^/r.u.): violet=SOMO; green=HOMO'. Shift, +0.0 eV (undoped film); +5.1 eV (doped film). (**c**) Measured UPS band centre energies plotted against the solid-state ionization potentials of the undoped TAF copolymers for undoped and strongly hole-doped TAF:SbF_6_. Band labelling follows band sequence. Lines are linear regression fits. The HOMO'-1 band is also the SOMO band.

We examined the doping-induced evolution of the VB structure of the TAF copolymers. Their weakly dispersive HOMO and HOMO-1 bands make tracking possible, different from the homopolymer OSCs^30^,^31^. 20-nm-thick photo-crosslinked TAF copolymer films were hole doped with NOSbF_6_ and then compensated to different DLs in the N_2_ glovebox by washing with commercial-grade nitromethane. The film DL was established by X-ray photoemission spectroscopy (XPS). Figure 2a shows a set of curve-fitted C1s, N1s and F1s core-level spectra collected from mTFF:SbF_6_ films. The C1s binding energy (BE) appears at 289.4 eV versus VL, N1s at 404.65 eV for the undoped amine site and F1s at 693.1 eV for the –CF_3_ moiety. Doping introduces an additional F1s emission at 690.5 eV from SbF_6_^−^ and broadens the N1s spectrum towards higher BE due to the doped amine (aminium) site (denoted N^+^, 405–409 eV). DL was independently evaluated by the ratio of counter-anion concentration to total amine concentration, that is, DL=[SbF_6_^−^]/[*N*_tot_] where [ · ] is the photoemission cross-section corrected intensity; and also by the curve-fitted aminium ratio, that is, DL=[N^+^]/ [*N*_tot_]. Both estimates agree to better than ±10% relative, for 0.1≲DL≲1.0 *h*^+^/r.u. This confirms the polarons are indeed well behaved and reside at the N sites. Furthermore, the invariance of the C1s and F1s BEs across different hole-doped TAFs (variation<0.1 eV) confirms that the different *ϕ*s arise from a change in the inherent electrochemical potential of the holes, rather than the surface dipole due to ionic layering^35^ or surface segregation^8^.

Figure 2b shows the VB evolution for the set of TAF:SbF_6_ films. The frontier 2 eV comprises two bands, labelled HOMO and HOMO-1. Hole doping produces complex changes accompanying the expected FL shift towards the VB edge. The intensity of the original HOMO band decreases, but new intensity emerges in the band valley, and the HOMO-1 band broadens considerably. At full doping of 1 *h*^+^/r.u., the band shape indicates two broad overlapping bands. The FL is ultimately pinned *ca*. 250 meV outside the band edge, as observed also in other n- and p-doped OSCs^36^,^37^,^38^,^39^, perhaps due to incomplete relaxation of the final state in UPS. The band structure evolution here reveals changes in the DOS that were previously obscured.

**Figure 3:**
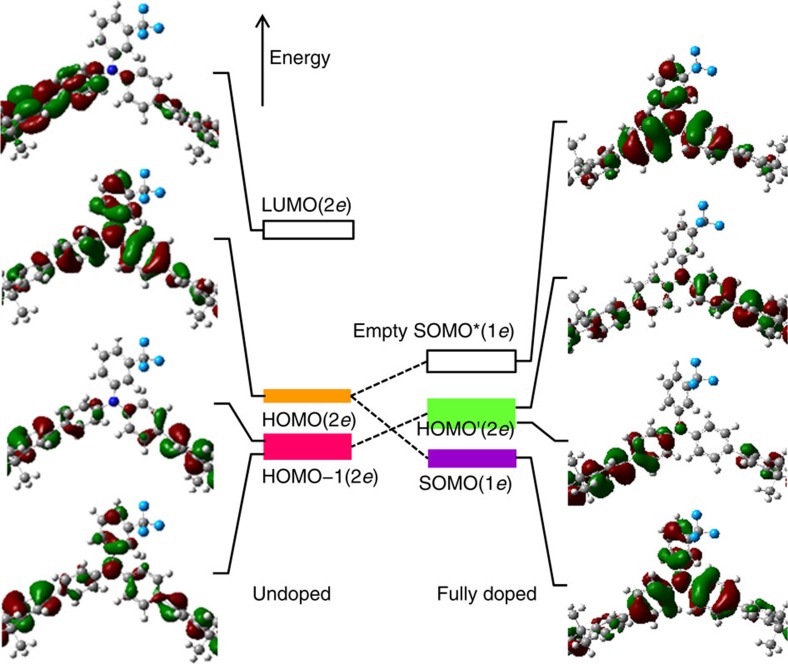
DFT-computed molecular orbital wavefunctions. Illustrative molecular orbital (MO) wavefunctions of the LUMO, HOMO and HOMO-1 bands for a repeat unit of the undoped (left) and fully hole*-*doped (right) mTFF polymer. Atoms: dark blue (nitrogen), grey (carbon), white (hydrogen) and light blue (fluorine). The LUMO, HOMO and HOMO-1 of the undoped polymer are each two-electron bands. These bands are weakly dispersive (computed transfer integral *t*: 2*t*≈60 meV for HOMO and 160 meV for HOMO-1). The HOMO' of the doped polymer is a two-electron band, but the SOMO and empty SOMO* are each one-electron bands split by the Hubbard gap, derived from spin orbitals. The right panel is shifted with respect to the left, depending on Coulomb interactions with counter-ions and other carriers.

To assign these changes, we performed density functional theory (DFT) calculations using CAM-B3LYP hybrid functional^40^, 6-311G(*dp*) basis, on a trimer model to clarify band parentage at the top of the VB (see Methods: Quantum chemical calculations). The wavefunctions of the central portion of the trimer were extracted to represent the repeat unit (Fig. 3). For the undoped TAF, the calculations show that the HOMO band (2*e*^−^/r.u.) derives from the ‘lone-pair' *π*-MO on the triarylamine unit, whereas the HOMO-1 band (2*e*^−^/r.u.) derives from the *π*-MO on the phenylene sequence between the N atoms (Fig. 3). This latter band is split by symmetric and antisymmetric combinations, which amount to 0.1 eV in TFB, increasing to 0.25 eV in TFBF. These features are in agreement with experiment. The computed band centres are marked in orange (for HOMO) and magenta (HOMO-1) in Fig. 2b. The CAM-B3LYP functional produces an error in these gas-phase calculations that coincides with solid-state relaxation effects, which reproduces experimental values without further correction. These assignments are confirmed by band systematics. The experimental HOMO band shows a greater sensitivity to pendant-ring substitution than the HOMO-1 band. The band centre shifts with *I*_p_ at the rate of 0.94±0.05 eV per eV for HOMO versus 0.79±0.04 eV per eV for HOMO-1 (Fig. 2c). This confirms the HOMO band has the stronger triarylamine character.

Upon hole doping, the calculations reveal that the HOMO splits into a SOMO and an empty counterpart SOMO* (both are spin orbitals, each with 1 *e*^−^/r.u.), separated by a gap of 3.9 eV at this level of theory, independent of ring substitution. This splitting is a manifestation of the Hubbard gap. Furthermore, Coulomb charging of the orbitals downshifts their energies, partly mitigated by Coulomb repulsion with the anion. As a consequence, the SOMO band is downshifted below the original HOMO-1 band, which now becomes the new HOMO' band, with the empty SOMO* band lying just above it. This band reordering is consistent with the band shape evolution. The splitting in the HOMO' band increases by 30−60%, which contributes to broadening. The new bands are marked in green (HOMO') and violet (SOMO, also called HOMO'-1) in Fig. 2b. In particular, the difference between the calculated HOMO' and SOMO positions matches experiment well, as shown by second-order derivative spectra (Supplementary Fig. 2). Furthermore, the band positions after a fixed rigid shift to accommodate Coulomb, polarization and other solid-state effects produces good agreement with experiment. This reversal of orbital ordering results in the HOMO' band exhibiting a lower sensitivity to ring substitution than the HOMO'-1 band, which is experimentally observed: 0.32±0.02 eV per eV versus 0.42±0.02 eV per eV (Fig. 2c). Antiferromagnetic coupling of spins on adjacent N sites is expected to lead to a spinless SOMO band.

### Revised polaron band model

**Figure 4:**
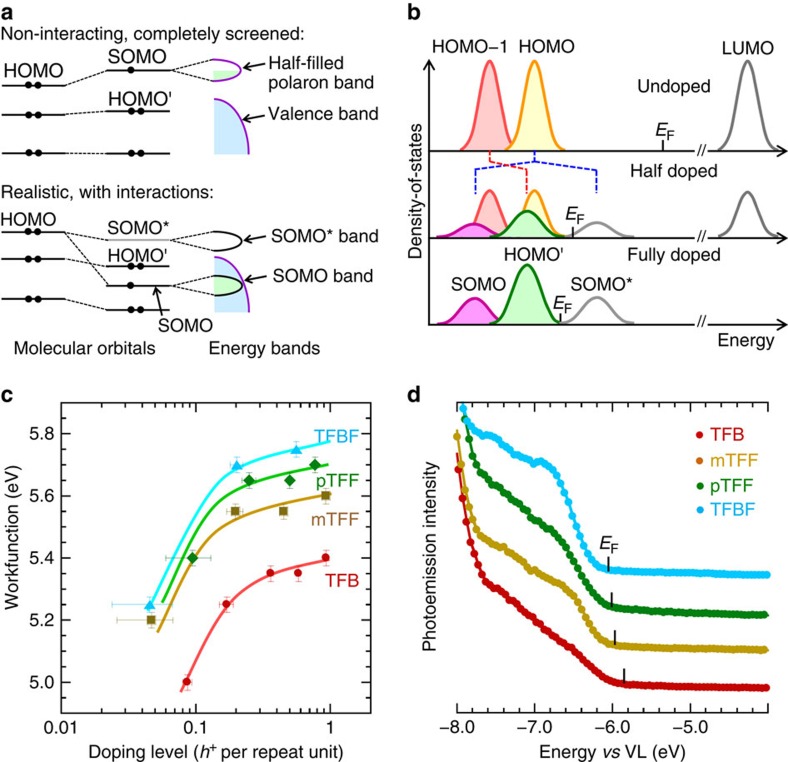
Evolution of DOS and workfunction dependence on DL. (**a**) Schematic drawing of valence molecular orbital energy levels before and after hole doping for the standard polaron band model and the revised polaron band model taking into account interactions, for an OSC that can accommodate two polarons on the same chain or in one repeat unit. The lowering of SOMO below the original HOMO is generic. (**b**) Dynamic DOS model applied to hole-doped TAF. The bands are colour coded as in Fig. 2c: filled bands are shaded, empty bands are clear. The depletion of the orange HOMO band leads to growth of the violet SOMO band and the uncoloured SOMO* band. Concomitantly, the red HOMO-1 band shifts and broadens to give the green HOMO' band. The two bands in the simplified two-band model (see text) refer to the SOMO* band and combined HOMO + HOMO' bands flanking *E*_F_. (**c**) Plot of UPS workfunction against hole DL. Curves are drawn as guides to the eye. Separate global fitting was performed in the heavily doped (0.3−1.0 *h*^+^/r.u.) and lightly doped (<0.2 *h*^+^/r.u.) regimes. The limiting slope drawn at high DLs is 95 meV per decade. Typical errors: measured vacuum workfunction, ±0.025 eV; DL, ±0.01 to ±0.04 (due to uncertainties in the extraction method, in particular at low DL). (**d**) Ultraviolet photoemission VB spectra for heavily hole-doped triarylaminium-based polymers with BArF^−^ as counter-anion. DL: 0.85 (TFB), 0.65 (mTFF), 0.7 (pTFF) and 0.4 (TFBF).

Therefore, the hole-doped SOMO band actually lies below the original HOMO band of the OSC. For organic semiconductors with large repeat units, the SOMO may even lie below a new HOMO', as the TAF copolymers show here. This is also the case for organic semiconductors with multiple small repeat units doped in the polaron regime. Addition of a hole to the HOMO generates a SOMO downshifted and buried beneath the new HOMO'. This explains the well-known phenomenon that doping of long chains produces multiple polarons before reaching the onset of bipolaron generation. For example, oxidation of the dodecithiophene monocation in solution produces not a bipolaron but a polaron pair^41^. The revised model is shown in Fig. 4a and compared with the standard model. There is a fundamental change in the nature and ordering of the orbitals and bands in the frontier region. So long as the hole-doped polymer OSC has not reached the bipolaron limit, its SOMO band must lie below the VB edge. Thus there is no discrete hole polaron state in the gap despite its frequent depiction in the literature. For the case of electron doping, a filled SOMO band is expected to emerge below and an empty SOMO* band above the conduction band edge. Thus a discrete electron polaron state can exist in the gap. 

### Dependence of workfunction on doping level

One important consequence of this model is the FL of the hole-doped OSC is determined not by the highest filling level in a partially filled polaron band, but by the thermal equilibration of electrons and holes at the conjunction of the filled valence and empty SOMO* bands. The *ϕ* is given by the FL lying at this conjunction. As the frontier DOS structure is not static, but evolving continually with doping due to emergence and shift of the HOMO' and SOMO* bands, the DOS model for the dependence of *ϕ* on DL has to consider these band structure changes.

The simplest DOS model for the hole-doped TAF copolymers is sketched in Fig. 4b. Assuming a rudimentary two-band model (Supplementary Fig. 3), that is, one band for holes and the other for electrons, each with Gaussian distribution and identical width, we show in Supplementary Note 1 that the Fermi–Dirac integral gives: 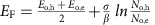, where *E*_o,h_ and *E*_o,e_ are the centre energies for the hole and electron bands, respectively, *σ* is the Gaussian width, *β* is a numerical factor that depends on band separation, band width and temperature, and *N*_o,h_ and *N*_o,e_ are the integrated DOS in the hole and electron bands, respectively. This equation holds under the usual conditions that both 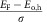 and 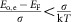, which are satisfied here, and requires only that the frontier portions of the bands are Gaussian-like. For the TAF copolymers, *E*_o,e_−*E*_o,h_≈1.1 eV, as estimated from the *P*_1_ absorption, *σ*≈225 meV and k*T*=25 meV. This gives *β*≈5.5 (Supplementary Table 1). The model then predicts for the heavily doped regime a slope of 95 meV shift in *E*_F_ per decade of doping. This is larger than the 60 meV per decade shift from classical semiconductor band theory. Measurements of the dependence of *ϕ* on DL for TAF:SbF_6_ are shown in Fig. 4c. The average slope is indeed 95 meV (±20 meV) per decade of doping, for 0.3≲DL≲1.0 *h*^+^/r.u. This quantitative agreement provides evidence for the role of an evolving SOMO*. Although the direction of shift of *ϕ* with DL may have been deduced from the Nernst equation, its actual dependence requires consideration of the evolving band structure. These results further confirm that *ϕ* is well defined in the heavily doped regime, which is beneficial for contact formation. 

### Dependence of workfunction on counter-ion

Another important consequence of the revised model is its emphasis on the role of Coulomb interactions on band energetics and hence work function. This has previously been suggested by spectator ion effects^33^. The central idea is the Coulomb shift of the orbitals and bands depends on counter-ion and other charge distances. This is directly suggested by the experimental shift in the VB spectra leading to an increase in *ϕ* by 0.3−0.4 eV when the counter-anion is changed from SbF_6_^−^ to BArF^−^ (compare Figs 2b and 4d).

The Coulomb interaction energy can be computed for a simple structural model of the doped TAF copolymers. In the lowest approximation, this energy depends only on two distances: the average hole…hole distance 〈*d*_hh_〉 and the average hole…counter-anion distance 〈*d*_ha_〉. Despite the absence of detailed structural information, these parameters can be deduced from Voronoi cell statistics and molecular modelling, as shown in Supplementary Tables 2 and 3 (see also Supplementary Note 2). Coulomb attraction results in the formation of ion clusters with close hole–anion contact pairs. These may be frustrated by steric effects, such as in highly asymmetric ionic liquids^42^. The shift of orbital energies due to Coulomb potential of the nearest anion at 〈*d*_ha_〉 can then be estimated by DFT calculations, taking into account possible perturbation of the MOs (Supplementary Fig. 4). Such perturbations turn out to be small for realistic 〈*d*_ha_〉 values. The Coulomb potential due to other holes and their associated anions can then be estimated through a Madelung factor in the point-charge approximation, which only depends on the 〈*d*_hh_〉 to 〈*d*_ha_〉 ratio. Detailed analysis is given in Supplementary Note 3 and Supplementary Fig. 5. The results show that the relevant band energies in TAF:BArF are destabilized, that is, downshifted, by 0.4 eV versus TAF:SbF_6_ (Supplementary Table 4). Despite the simplicity of this putative model, good agreement with experiment was obtained, confirming the primary role of Coulomb interactions. This effect arises chiefly from the destabilization of the orbital energies by the counter-ion, which is only partly offset by the Madelung sum. Thus, the use of large counter-ions or equivalently the frustration of their close approach to the doped carriers, whether holes or electrons, can produce large favourable shifts in the workfunction.

### Ohmic hole contacts to deep *I*_p_ semiconductors

**Figure 5:**
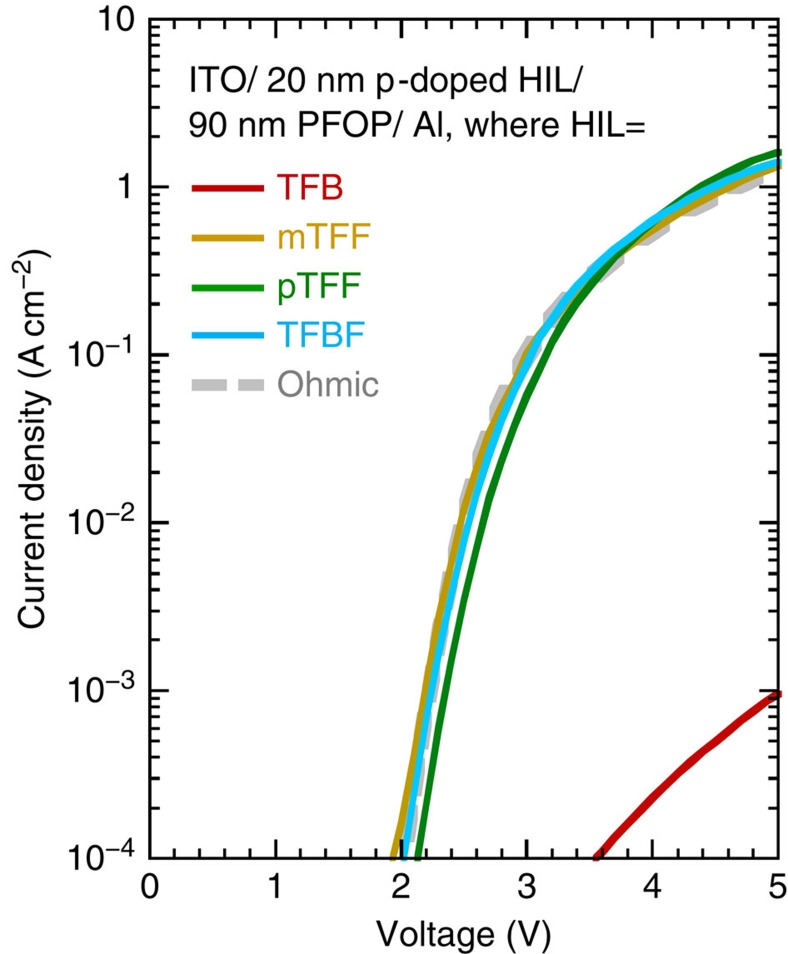
Current–voltage characteristics of diodes with different TAF:SbF_6_ HILs. The grey dashed line gives the computed characteristic for ohmic hole contact with parameters: hole mobility, 5 × 10^−4^ cm^2^ V^−1^ s^−1^; built-in potential, 2.5 V; hole-contact carrier density, 2 × 10^17^ cm^−3^.

Finally, we show that these ultrahigh workfunction materials can provide useful ohmic hole injection^43^ into semiconductors with large *I*_p_ beyond the reach of poly(3,4-ethylenedioxythiophene): poly(styrenesulfonic acid) and sulfonated poly[3-(2-(2-methoxyethoxy)ethoxy)thiophene-2,5-diyl]: poly(4-hydroxystyrene). We employ poly(9,9-bis(4-octylphenyl)fluorene-2,7-diyl) (PFOP, *I*_p_=5.8 eV) as benchmark OSC with a large *I*_p_^3^. It has a suitably well-behaved and non-dispersive hole mobility. Figure 5 shows the current–voltage (*JV*) characteristics of hole-only diodes: indium-tin oxide (ITO)/HIL/PFOP/Al. These were fabricated with different TAF:SbF_6_ as model HILs, spin-cast and film-doped to give DL≈0.5−0.7 *h*^+^/r.u., on ITO-glass substrates. The diodes with doped mTFF, pTFF and TFBF show practically identical *JV* characteristics that are three orders of magnitude higher than diodes with doped TFB. To check that the ohmic regime has been reached, we computed the *JV* characteristic for an ideal ohmic injection contact, using standard drift–diffusion equations following Blom and co-workers^44^. The space-charge-limited-current hole mobility of PFOP is 5 × 10^−4^ cm^2^ V^−1^ s^−1^. The excellent match between experimental and computed characteristics confirms that doped mTFF and beyond indeed provide ohmic hole injection into PFOP. To achieve space-charge-limited current density, a threshold carrier density at the contact of a few 10^11^ cm^−2^ is required, which was found also in subgap electroabsorption measurements^45^. This condition is met when *ϕ* approaches within 0.2 eV of the *I*_p_ of PFOP. At the same time, ohmic contact with the ITO electrode is also achieved due to large carrier density in the HIL^46^.

In summary, the discovery of hole-doped TAFs with workfunctions up to 6.0 eV shows that the triarylaminium core is viable for high inherent workfunctions. This opens the path for the development of additive ambient solution-processed HILs that can inject into the most demanding semiconductors in both top and bottom configurations. Immobilization of the counter-anion is key to further improving the stability of these heavily-doped materials for general multilayer solution processing. Investigation of these hole-doped conducting polymers has in turn revealed the key roles of the long-neglected Hubbard and Madelung interactions in determining band energetics of the polaron. These interactions fundamentally change the nature and ordering of orbitals (and bands) at the frontier edges, and are required to explain the observed counter-ion size effect and doping effect on workfunction. The corollaries for electron-doped systems can be readily derived. The recognition that these interactions need to be included even in the minimal description of polaron band energetics resolves longstanding anomalies in the literature of doped OSCs.

## Methods

### p-Doping

The TAF copolymers were obtained from CDT/Sumitomo Chemical Co. or synthesized in-house following established Suzuki coupling procedures. The undoped TAF copolymer solutions (10 mM r.u., anhydrous chloroform) were reacted with 1.1 mol equiv of NOSbF_6_ (30 mM, anhydrous chloroform) to give fully p-doped polymer solutions with SbF_6_^−^ as counter-anion. This was ascertained by electronic spectroscopy of the solutions, which showed full development of the *P*_1_ and *P*_2_ polaron transitions at 1.1 and 2.5 eV, together with bleaching of the neutral *π*→π* band at 3.2–3.45 eV (TFB–TFBF copolymers). The p-doped TAF polymers were then purified by precipitation with diethyl ether and redissolution in chloroform or nitromethane, and films were then fabricated by spin-casting or printing in the N_2_ glovebox. To exchange SbF_6_^−^ with BArF^−^, the p-doped TAF copolymer solutions were mixed with 1.0 mol equiv NaBArF in anhydrous chloroform to precipitate NaSbF_6_, which was filtered off. No change was found in the electronic spectra of the solutions, so the DL was not degraded by this procedure. Thin films were then spin-cast in a N_2_ glovebox. For some applications, it may be convenient or desirable to p-dope thin films of the TAF copolymers after they were first deposited. In a typical procedure, 20-nm-thick films of the TAF copolymers with 6.5 mol/mol% of ethylene bis(4-azido-2-isopropyl-3,5,6-trifluorobenzoate) were formed on Au-coated Si, ITO or glass substrates. These films were photo-cross-linked at 254-nm wavelength^27^ and then contacted with a dilute solution of NOSbF_6_ in anhydrous propylene carbonate (10−50 mM) on a spinner in the glovebox (*p*O_2_, *p*H_2_O<1 p.p.m.). The workfunctions of these film-doped TAF copolymers were found to be similar to those of solution-doped materials at the same DL. The absence of chemical degradation was confirmed by complete reversibility of hole-doping on compensation with a mild reductant such as 7,7,8,8-tetracyano-1,4-quinonedimethane.

### UPS and XPS spectroscopies

UPS and XPS were performed on the films in this sequence in an ESCALAB UHV chamber equipped with an Omicron EA 125 U7 hemispherical electron energy analyser at a base pressure <10^−9^ mbar. Film samples were mounted in a N_2_ glovebox, transported to the analyser station in hermetically sealed N_2_ bags and loaded into the UHV chamber without exposure to ambient air. UPS was excited using He I radiation (21.21 eV) with minimal He II background. XPS was excited using MgKα X-rays (1253.6 eV). The photoemission normal to the film surface was collected and analysed at a resolution of 50 meV and pass energy of 5 eV for UPS, and a resolution of 0.7 eV and pass energy of 20 eV for XPS. To determine the XPS atomic stoichiometries, integrated core-level intensities were corrected with empirical atomic sensitivity factors. The (small) contributions from the cross-linker to both N1s and F1s photoemission were taken into account.

### Quantum chemical calculations

Electronic structures of the undoped TAF copolymers and their fully doped states were computed at the DFT/CAM-B3LYP/6-311G(*dp*) level. The CAM-B3LYP hybrid functional is an improved long-range-corrected hybrid functional that we have found to give better agreement than the older B3LYP hybrid functional with experimental *I*_p_s for a variety of *π*-conjugated organic molecules. Validation calculations were also performed on triphenylamine: vertical *I*_p_: 6.43 eV (calc), 7.00 eV (expt); adiabatic *I*_p_: 6.77 eV (calc) and 6.80 eV (expt). To avoid possible artefacts due to periodic boundary conditions, calculations were performed on a truncated trimer, which was sufficient to model the polymer at acceptable computational costs, owing to the large size of the repeat unit. The trimer were geometrically optimized by the PM6 Hamiltonian separately for both undoped and fully hole-doped states. Both have propeller-like twisting of the aryl rings about the nitrogen atom as expected. Pristine state is pyramidal at the N site: ∠CNC 116°; CN 1.46 Å. Hole-doped state is planar at the N site: ∠CNC 120°; CN 1.43 Å; ring-tilt angle relative to CNC plane, 35°. The calculations confirmed that the frontier molecular orbital wavefunctions are indeed relatively localized, in agreement with electronic spectroscopy and XPS results, which validates the choice of trimers as models for the polymers.

### Fabrication of hole-only diodes with PFOP as model semiconductor

Doped and purified TAF polymers in nitromethane solutions were prepared by solution-state doping as described above with 1.1 mol equiv of NOSbF_6_. 20-nm-thick HILs were spin-cast in the N_2_ glovebox from these solutions onto Standard Clean 1-treated ITO substrates. PFOP was spin-cast from toluene solution over the HILs in the glovebox to give 125-nm-thick films. 120-nm-thick Al was thermally evaporated through a shadow mask at a base pressure of 10^−7^ Torr to give the cathode for eight 4.3-mm^2^ pixels on each substrate. The current–voltage characteristics were collected on a probe station in the glovebox using a Keithley 4200 semiconductor parameter analyser.

### Data availability

The data supporting the findings of this study are available within the article and its Supplementary Information files.

## Additional information

**How to cite this article:** Png, R.-Q. *et al*. Madelung and Hubbard interactions in polaron band model of doped organic semiconductors. *Nat. Commun.* 7:11948 doi: 10.1038/ncomms11948 (2016).

## Supplementary Material

Supplementary Information Supplementary Figures 1-5, Supplementary Tables 1-8, Supplementary Notes1-4
