# Supplementary material for: Madelung and Hubbard interactions in polaron band model of doped organic semiconductors
Source: Nat Commun. 2016 Sep 1;7:11948. doi: 10.1038/ncomms11948 (PMC5025745; doi:10.1038/ncomms11948)
Supplement: Supplementary Information — Supplementary Figures 1-5, Supplementary Tables 1-8, Supplementary Notes1-4 [file ncomms11948-s1.pdf]

Supplementary Figure 1

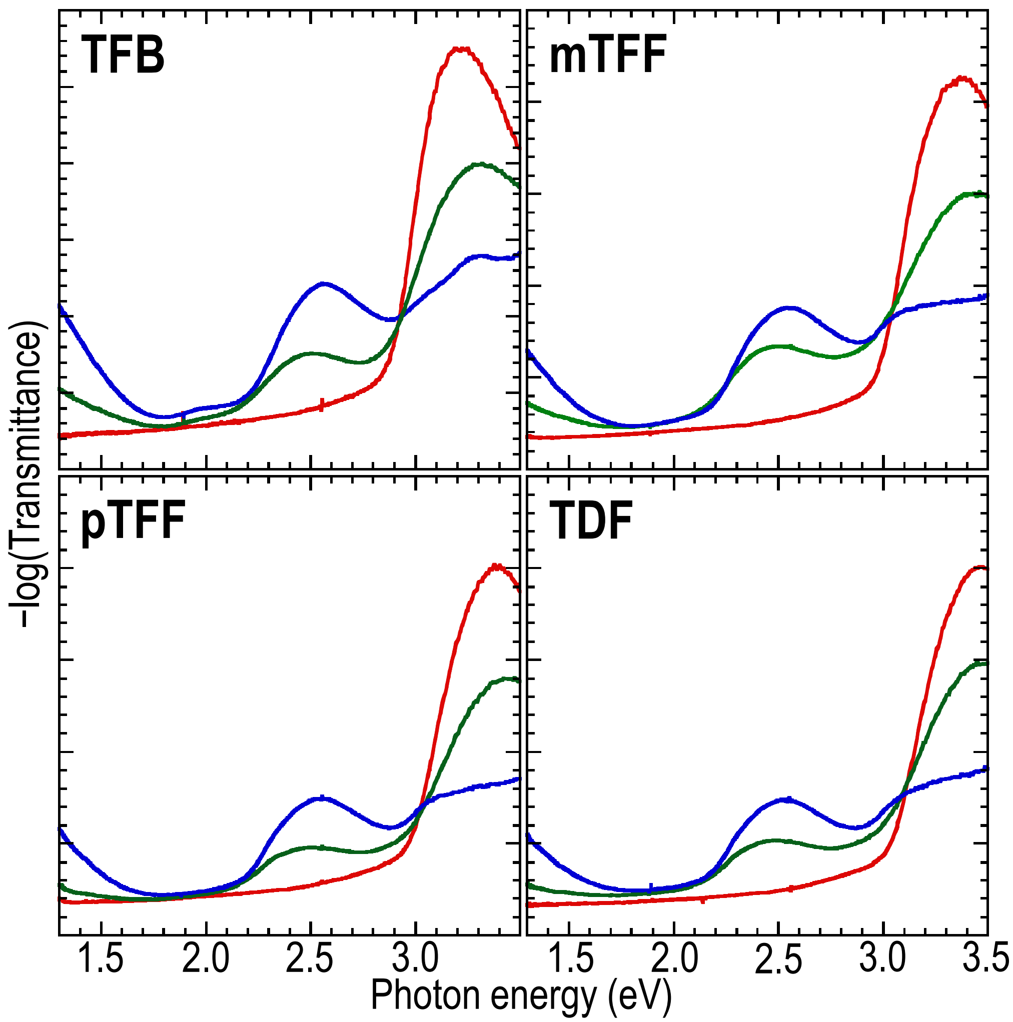

Supplementary Figure 1. Electronic absorption spectra of *p*-doped TAF copolymer thin films. Doping level: 1.0 h^+/ r.u. (blue), 0.4−0.6 h^+/ r.u. (green), undoped (red). Substrate, fused silica. The integrated intensity of the emergent polaron P_2 band at 2.5 eV scales linearly with the bleaching of the $\pi \rightarrow \pi^*$ band at 3.2−3.45 eV, confirming near independent behavior of the hole-doped nitrogen sites. The intensity of the P_2 band also scales linearly with doping level measured by XPS, confirming uniform doping through the entire film thickness.

Supplementary Figure 2

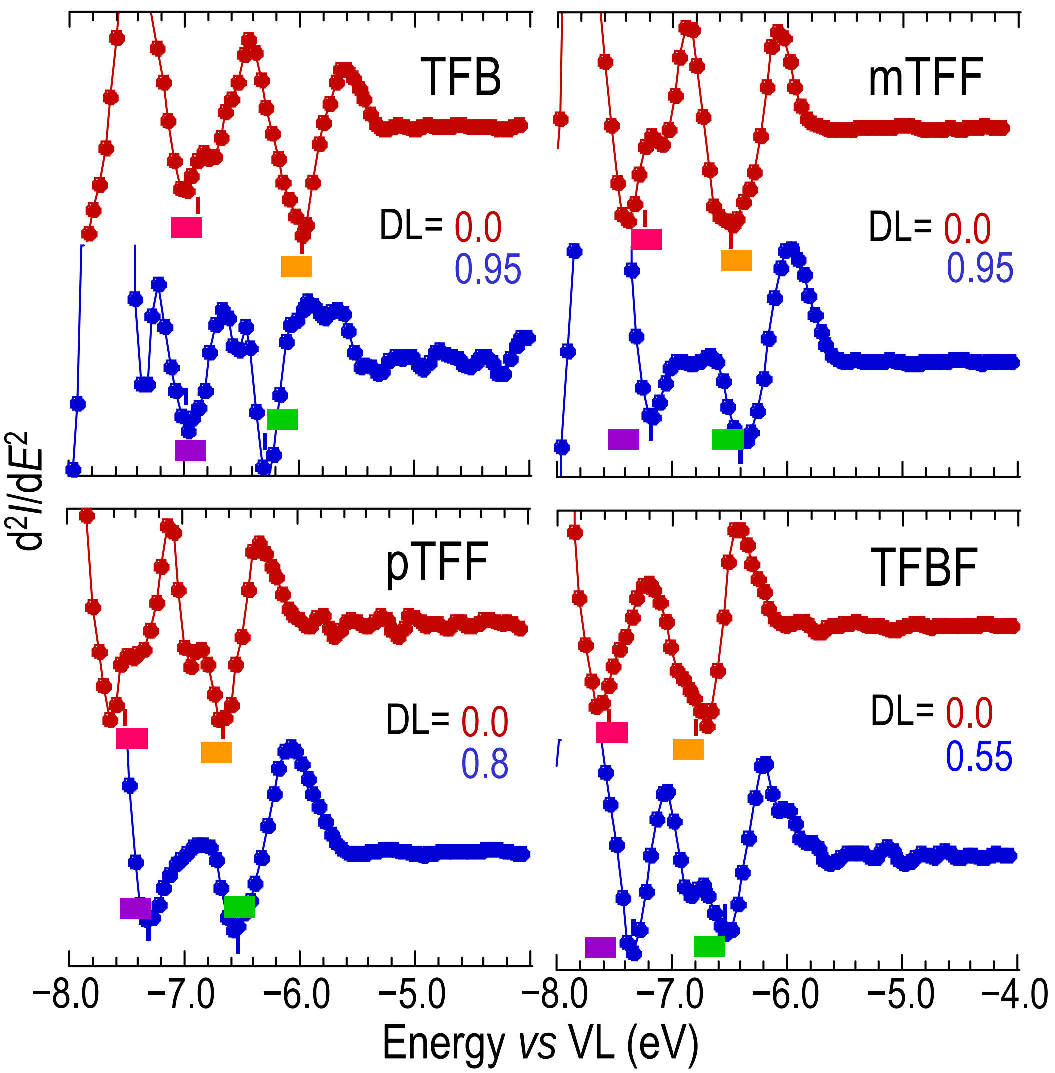

Supplementary Figure 2. Second-derivative ultraviolet photoemission spectra plotted against energy measured from VL for undoped and doped films. The data was fitted to a smoothing spline function (smoothing factor, 1) with standard deviation corresponding to experimental noise. The first derivative was obtained numerically. This was fitted to a smoothing spline function. The second derivative was obtained numerically and presented here. Peak positions were marked based on peak maxima. For DL = 0.0, the HOMO–1 band position was marked as the arithmetic mean of the symmetric and anti-symmetric combination components. These doublets are not resolved in the HOMO band of the doped spectrum because of band broadening. The computed mean band positions from Fig. 2b are superposed for comparison.

Supplementary Figure 3

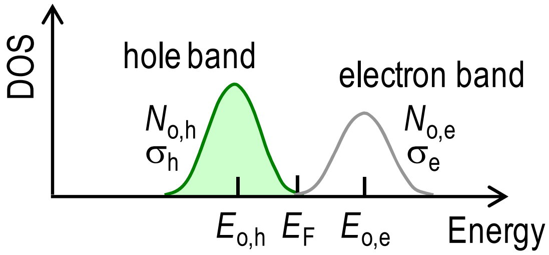

Supplementary Figure 3. Schematic of dynamic DOS model

Supplementary Figure 4

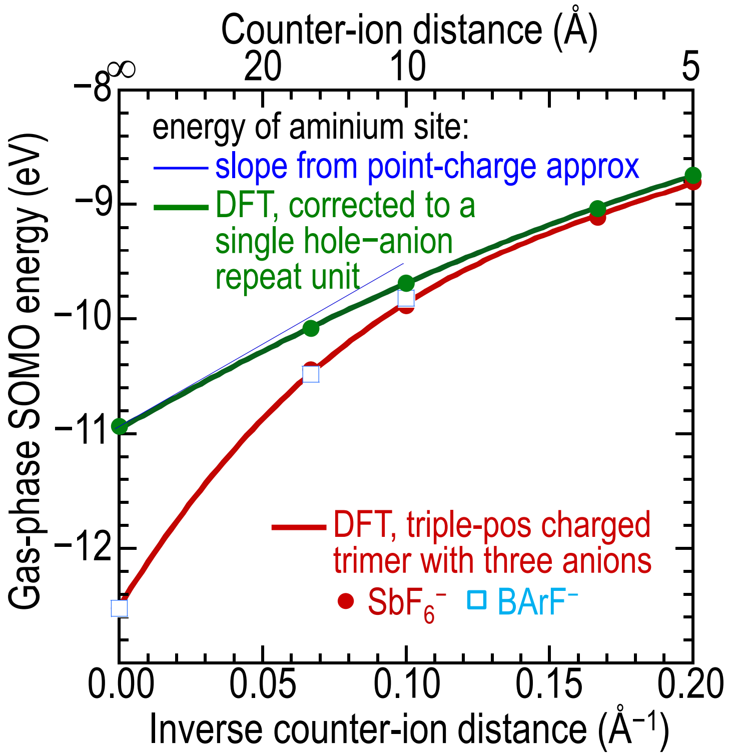

Supplementary Figure 4. Dependence of SOMO energy on counter-ion distance. Plot of gas-phase energy of the SOMO of a repeat unit of a fully hole-doped mTFF repeat unit as a function of the inverse distance to its counter-anion, computed at the DFT/ CAM-B3LYP/ 3-211G(*dp*) level. This was modeled as a triple-positively charged mTFF trimer with three counter-anions positioned directly over the nitrogen atoms at various distances perpendicular to the local triarylaminium plane (red line), and corrected by subtracting the coulomb interaction of the central nitrogen site with each of the two end hole−counter-anion pairs (green line). The limiting behavior at infinite hole−counter-anion separation where the point-charge approximation holds (14.4 eV Å) is also shown (blue line). Significant deviation begins at a counter-anion distance of 15 Å due to the extended size of the SOMO wavefunction.

Supplementary Figure 5

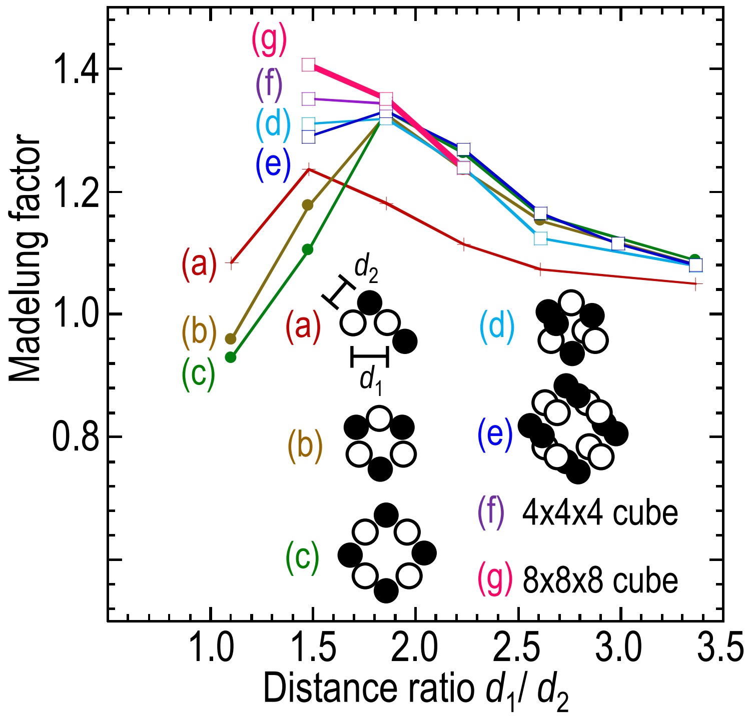

Supplementary Figure 5. Computed Madelung factors for constraint 1:1 ion clusters. The Madelung factor is defined as the ratio of the total electrostatic stabilization energy per cation in the ion cluster ($u_{el,cat}$) to the electrostatic stabilization energy of an isolated cation−anion pair at closest approach ($u_{el,pair}$): $M = u_{el,cat} / u_{el,pair.}$ 1:1 Neutral ion clusters comprising two (i.e., ion quartet) to 512 ion pairs in various high-symmetry cation sub-lattices were considered. The sub-lattice parameter was systematically dilated to simulate geometric constraints on the holes. The positions of the anions were then optimized to the cation sub-lattice. Na^+ and Cl^- ions were employed as models to impose realistic "hard" sphere size-exclusion potentials. The simulations were performed using molecular mechanics (MM2) force fields. The Madelung factor obtained was then plotted against the d_1/ d_2 ratio, where d_1 is the nearest-neighbor cation...cation distance, and d_2 is the nearest-neighbor cation...anion distance. For comparison, the M values for NaCl and CsCl lattices fall within1.75−1.76. The limit of M for large d_1 / d_2 is unity, as expected. The inset shows representative structures of the ion cluster models: (open circles) cations, (filled circles) anions.

Supplementary Table 1

No	$E_{o,e} - E_{o,h}$ (eV)	σ (meV)	kT (meV)	β
1	1.1	225	25	5.5
2	1.1	200	25	5.9
3	1.3	225	25	6.2
4	1.3	200	25	6.7

Supplementary Table 2

Supplementary Table 2. Repeat unit volume (v_{ru}) and mean nearest-neighbor hole…hole distance ($\langle d_{hh} \rangle$) of fully *p*-doped polymers used in the study.

Polymer	v_{ru} (cm^3)	$\langle d_{hh} \rangle$ (Å)
TFB: SbF_6	1.26×10^{-21}	12.1
TFB: BArF	1.93×10^{-21}	14.0
mTFF: SbF_6	1.20×10^{-21}	11.9
mTFF: BArF	1.90×10^{-21}	13.9
pTFF: SbF_6	1.20×10^{-21}	11.9
pTFF: BArF	1.90×10^{-21}	13.9
TFBF: SbF_6	1.26×10^{-21}	12.1
TFBF: BArF	1.96×10^{-21}	14.0

Supplementary Table 3

Supplementary Table 3. Average closest approach distances of counter-anions to triphenylaminium ion.

Counter-anion	Polar $\langle d_{ha} \rangle$ (Å) [a]	Equatorial $\langle d_{ha} \rangle$ (Å) [b]	Method
SbF_6^-	4.57 ± 0.05	6.1 ± 0.2	PM3, MM2
$BArF^-$	7.0 ± 0.3	7.2 ± 0.5	MM2

Footnotes:

Center-to-center distance between ion and nitrogen atom for:

[a] polar closest approach above/ below molecular plane; and

[b] equatorial closest approach in between tilted phenyl rings.

with standard errors given for eight optimized sample configurations.

Supplementary Table 4

Supplementary Table 4. Computed shift in orbital energetics.

**Counter-anion**	**⟨d_{hh}⟩ (Å)**	**⟨d_{ha}⟩ (Å)** [a]	*M*	**u_i (eV)**	**$\sum_j u_{el,j}$** [b] **(eV)**	**$u_i + \sum_j u_{el,j}$ (eV)**
SbF_6^-	12	4.6 (pol)	1.15	−8.63	−0.19	−8.82
		6.1 (eq)	1.30	−9.05	−0.28	−9.33
$BArF^-$	14	7.0 (pol)	1.30	−9.23	−0.25	−9.48
		7.2 (eq)	1.32	−9.28	−0.26	−9.54

Footnotes:

[a] pol = polar closest approach, eq = equatorial closest approach.

[b] computed with ε_r = 2.5.

Supplementary Table 5

Supplementary Table 5. Estimation of the repeat unit molar volume (v_{ru}) of mTFF by additive group molar volume contribution method.

**Group**	**Formula**	**$V_{a,i}$ (cm^3 mol^{-1})**	**Quantity**	**Sub-total (cm^3 mol^{-1})**
phenylene	$-C_6H_4-$	65.5	5	327.5
methylene	$-CH_2-$	16.4	14	229.6
methyl	$-CH_3$	21.9	2	43.8
Trifluoromethyl	$-CF_3$	34.1	1	34.1
tetrasubstituted carbon	C	5.0	1	5.0
trisubstituted nitrogen	N	6.4	1	6.4
**v_{ru}**				646.4

Supplementary Table 6

Supplementary Table 6. Repeat unit volumes (V_{ru}) of pristine polymers in this study estimated by group contribution method.

Polymer	V_{ru} (cm^3)	ρ (g cm^{-3})
TFB	1.13×10^{-21}	1.00
mTFF	1.07×10^{-21}	1.06
pTFF	1.07×10^{-21}	1.06
TFBF	1.13×10^{-21}	1.11

Supplementary Table 7

Supplementary Table 7. Molecular volume of $BArF^-$ computed by group contribution method.

Group	Formula	$V_{a,i}$ (cm^3 mol^{-1})	Quantity	Sub-total (cm^3 mol^{-1})
phenylene	$-C_6H_4-$	65.5	4	262.0
trifluoromethyl	$-CF_3$	34.1	8	272.8
tetrasubstituted boron	B	6.0	1	6.0
hydrogen	−H	4.8	−4	−19.2
steric crowding @ tetrahedral center	--	−42.8	1	−42.8
**molecular volume**				478.8

Supplementary Table 8

Supplementary Table 8. Molecular volume of tetraphenylmethane estimated by group contribution method.

Group	Formula	$V_{a,i}$ (cm^3 mol^{-1})	Quantity	Sub-total (cm^3 mol^{-1})
phenylene	$-C_6H_4-$	65.5	4	262.0
tetrasubstituted carbon	C	5.0	1	5.0
hydrogen	−H	4.8	4	19.2
steric crowding @ tetrahedral center	--	−42.8	1	−42.8
**molecular volume**				243.4

Supplementary Note 1

The dynamic density-of-states model

The Fermi-Dirac integral for carrier occupation in a band with a gaussian density-of-states (DOS) distribution is given by (see Supplementary Figure 3)

$\int f_{\mathrm{FD}}(\hat{E}_i)G(\hat{E}_i)d\hat{E}_i,$

where $f_{\mathrm{FD}}(\hat{E}_i)$ is the Fermi−Dirac distribution function, $G(\hat{E}_i)$ is the relevant gaussian DOS function, and i denotes electron or hole.

For an electron band: $f_{\mathrm{FD}}(\hat{E}_{\mathrm{e}}) = (1 + \exp\frac{\hat{E}_{\mathrm{F,e}}}{\hat{s}_{\mathrm{e}}})^{-1}$, and $G(\hat{E}_{\mathrm{e}}) = \frac{N_{\mathrm{o,e}}}{\sqrt{2\pi}}\exp(-\frac{1}{2}\hat{E}_{\mathrm{e}}{}^2)$, where $\hat{E}_{\mathrm{F,e}} = \frac{E_{\mathrm{e}} - E_{\mathrm{F}}}{\sigma_{\mathrm{e}}}$ is the reduced Fermi energy for electrons in the electron band, $\hat{E}_{\mathrm{e}} = \frac{E_{\mathrm{e}} - E_{\mathrm{o,e}}}{\sigma_{\mathrm{e}}}$ is the reduced electron energy, σ_{e} is the standard gaussian width of the electron band, $E_{\mathrm{o,e}}$ is the center of the electron band, $\hat{s}_{\mathrm{e}} = \frac{\mathrm{k}T}{\sigma_{\mathrm{e}}}$ is the reduced thermal energy for electrons in the electron band, where $N_{\mathrm{o,e}}$ is the integrated DOS of the electron band.

For a hole band: $f_{\mathrm{FD}}(\hat{E}_{\mathrm{h}}) = (1 + \exp\frac{\hat{E}_{\mathrm{F,h}}}{\hat{s}_{\mathrm{h}}})^{-1}$, and $G(\hat{E}_{\mathrm{h}}) = \frac{N_{\mathrm{o,h}}}{\sqrt{2\pi}}\exp(-\frac{1}{2}\hat{E}_{\mathrm{h}}{}^2)$, where $\hat{E}_{\mathrm{F,h}} = \frac{E_{\mathrm{F}} - E_{\mathrm{h}}}{\sigma_{\mathrm{h}}}$ is the reduced Fermi energy for holes in the hole band, $\hat{E}_{\mathrm{h}} = \frac{E_{\mathrm{o,h}} - E_{\mathrm{h}}}{\sigma_{\mathrm{h}}}$ is the reduced hole energy, σ_{h} is the standard gaussian width of the hole band, $E_{\mathrm{o,h}}$ is the center of the hole band, $\hat{s}_{\mathrm{h}} = \frac{\mathrm{k}T}{\sigma_{\mathrm{h}}}$ is the reduced thermal energy for holes in the hole band, where $N_{\mathrm{o,h}}$ is the integrated DOS of the hole band.

Hemi-gaussian bands: The results developed here will hold provided the frontier portion of the DOS band is gaussian. This is because of the Fermi-Dirac integral is sensitive particularly only to this part of the band. In this case, $N_{\mathrm{o},i}$ has to be interpreted as the effective integrated DOS for the fitted gaussian to the hemi-gaussian band, $E_{\mathrm{o},i}$ is the center of the fitted gaussian, and σ_i as the standard width of the fitted gaussian.

Case I. For $\hat{E}_{\mathrm{F},i} > \hat{s}_i{}^{-1}$, where the distance from the E_{F} to the band center in units of width is larger than the width of the band in units of $\mathrm{k}T$, the integral I_i is well approximated by

$$lnI_i = lnN_{\mathrm{o},i} + \tfrac{1}{2}\hat{s}_i{}^{-2} - (\hat{E}_{\mathrm{F},i} - \hat{E}_i)\,\hat{s}_i{}^{-1}$$ Supplementary Equation 1

At thermal equilibrium: The number of electrons in the electron band equals the number of holes in the hole band. Assuming both $\hat{E}_{\mathrm{F,h}} > \hat{s}_{\mathrm{h}}{}^{-1}$ and $\hat{E}_{\mathrm{F,e}} > \hat{s}_{\mathrm{e}}{}^{-1}$,

$$lnN_{\mathrm{o,h}} + \frac{1}{2}\hat{s}_{\mathrm{h}}{}^{-2} - \hat{E}_{\mathrm{F,h}}\hat{s}_{\mathrm{h}}{}^{-1} = lnN_{\mathrm{o,e}} + \frac{1}{2}\hat{s}_{\mathrm{e}}{}^{-2} - \hat{E}_{\mathrm{F,e}}\hat{s}_{\mathrm{e}}{}^{-1} ,$$ Supplementary Equation 2

Assuming further that $\sigma_{\mathrm{h}} = \sigma_{\mathrm{e}} = \sigma$, we get

$$E_{\mathrm{F}} = \frac{\mathrm{k}T}{2} ln\frac{N_{\mathrm{o,h}}}{N_{\mathrm{o,e}}} + \frac{E_{\mathrm{o,h}} + E_{\mathrm{o,e}}}{2} .$$ Supplementary Equation 3

This result is analogous to that from classical band semiconductor theory.

Case II. For $\hat{E}_{\mathrm{F},i} < \hat{s}_i{}^{-1}$, where distance from the E_{F} to the band center in units of width is smaller than the width of the band in units of $\mathrm{k}T$, the integral I_i is well approximated by

$$lnI_i = ln\frac{N_{\mathrm{o},i}}{2} + (ln\frac{1}{2})(\hat{E}_{\mathrm{F},i} - \hat{E}_i) - \frac{1}{2}(\hat{E}_{\mathrm{F},i} - \hat{E}_i)^2 + \frac{3}{4}\,\hat{s}_i(\hat{E}_{\mathrm{F},i} - \hat{E}_i)^2 .$$ Supplementary Equation 4

At thermal equilibrium: The number of electrons in the electron band equals the number of holes in the hole band. Assuming both $\hat{E}_{\mathrm{F,h}} < \hat{s}_{\mathrm{h}}{}^{-1}$ and $\hat{E}_{\mathrm{F,e}} < \hat{s}_{\mathrm{e}}{}^{-1}$,

$$ln\frac{N_{\mathrm{o,h}}}{2} + (ln\frac{1}{2})(\hat{E}_{\mathrm{F,h}} - \hat{E}_{\mathrm{h}}) - \frac{1}{2}(\hat{E}_{\mathrm{F,h}} - \hat{E}_{\mathrm{h}})^2 + \frac{3}{4}\,\hat{s}_{\mathrm{h}}(\hat{E}_{\mathrm{F,h}} - \hat{E}_{\mathrm{h}})^2$$
$$= ln\frac{N_{\mathrm{o,e}}}{2} + (ln\frac{1}{2})(\hat{E}_{\mathrm{F,e}} - \hat{E}_{\mathrm{e}}) - \frac{1}{2}(\hat{E}_{\mathrm{F,e}} - \hat{E}_{\mathrm{e}})^2 + \frac{3}{4}\,\hat{s}_{\mathrm{e}}(\hat{E}_{\mathrm{F,e}} - \hat{E}_{\mathrm{e}})^2$$

Supplementary Equation 5

Assuming further that $\sigma_{\mathrm{h}} = \sigma_{\mathrm{e}} = \sigma$, we get

$E_{\mathrm{F}} = \frac{\sigma}{2} ln \frac{N_{\mathrm{o,h}}}{N_{\mathrm{o,e}}} \left(ln2 + \left(\frac{E_{\mathrm{o,e}} - E_{\mathrm{o,h}}}{\sigma} \right) \left(\frac{1}{2} - \frac{3}{4} \frac{\mathrm{k}T}{\sigma} \right) \right)^{-1} + \frac{E_{\mathrm{o,h}} + E_{\mathrm{o,e}}}{2}$. Supplementary Equation 6

This can be written as

$E_{\mathrm{F}} = \frac{\sigma}{\beta} ln \frac{N_{\mathrm{o,h}}}{N_{\mathrm{o,e}}} + \frac{E_{\mathrm{o,h}} + E_{\mathrm{o,e}}}{2}$, where $\beta = 2 \left(ln2 + \left(\frac{E_{\mathrm{o,e}} - E_{\mathrm{o,h}}}{\sigma} \right) \left(\frac{1}{2} - \frac{3}{4} \frac{\mathrm{k}T}{\sigma} \right) \right)$.

Supplementary Equation 7

The experimental situation conforms to case II. The value of β is not particularly sensitive to precise parameters for the bands, as can be seen in Supplementary Table 1.

Workfunction slope per decade of doping level: To compute the slope per decade, we approximate the evolution of $N_{\mathrm{o,h}}$ and $N_{\mathrm{o,e}}$ with the following functions:

$N_{\mathrm{o,h}} = N_{\mathrm{o}} * [2 * (1 - \vartheta) + 2 * \vartheta] = 2\ N_{\mathrm{o}}$, where ϑ is the doping level in h^+ per repeat unit, and first term in square brackets comes from bleaching of the HOMO, and the second term comes from shift of HOMO−1 to give HOMO'; and

$N_{\mathrm{o,e}} = N_{\mathrm{o}} * \vartheta$.

Hence $ln \frac{N_{\mathrm{o,h}}}{N_{\mathrm{o,e}}} = ln \frac{2}{\vartheta}$, and $ln \frac{N_{\mathrm{o,h}}}{N_{\mathrm{o,e}}} = ln2 - ln\vartheta = ln2 - 2.303\ lg\vartheta$. Supplementary Equation 8

For σ = 225 meV, β = 5.5, we get $\frac{dE_{\mathrm{F}}}{dlg\vartheta} = -2.303 \frac{\sigma}{\beta}$ = -95 meV per decade. The minus sign indicates the workfunction gets larger with doping, as expected and experimentally observed.

Supplementary Note 2

Estimation of mean values of the nearest-neighbor hole…hole (d_{hh}) and hole…counter-anion (d_{ha}) distances

The nearest-neighbor hole…hole distances (d_{hh}) are expected to exhibit a broad distribution from the shortest interchain distance given by eclipsed triarylaminium stacking, which is 4.6 Å (PM3 calculations), to the intrachain distance given by that between adjacent nitrogen sites separated by the fluorene-2,7-diyl–phenylene sequence, which is 18.2 Å (PM3 calculations). In the absence of structural order which prevents the extraction of atom−atom pair-correlation functions from X-ray diffraction, the required mean $\langle d_{hh} \rangle$ value can still be obtained from Voronoi cell statistics related to triarylaminium distances. The wavefunction of a hole is centered on a triarylaminium nitrogen atom. Let us assumed the position of this nitrogen atom is r_i. The hole Voronoi cell Vor(r_i) is defined to be the region of space no further from this nitrogen atom at r_i than to any other triarylaminium nitrogen atom at r_j, i.e., Vor(r_i) = { r : | $r - r_i$ | ≤ | $r - r_j$ | for all r_j in the material}. Thus the Voronoi cells partition space into polyhedral sub-spaces, one for each hole, where the polyhedron faces are equidistant to adjacent hole centers. The average volume of one of these Voronoi cells (v_{Vor}) is identical by definition to the average volume of a triarylaminium repeat unit including the counter-anion (v_{ru}), i.e., $v_{Vor} = v_{ru}$. Unfortunately the required v_{ru} values cannot be obtained from the usual X-ray scattering due to lack of long-range order, or from the flotation methods due to solvent swelling and/or de-doping, they can be accurately estimated using group contribution additivity methods. See the following Supplementary Note 4 for validation and application to these materials.

The average distance between nearest-neighbor holes is defined as the average distance between a hole center in one Voronoi cell and another hole center in the adjoining layer of Voronoi cells. For completely random packing of neighboring repeat units, the orientation of adjoining Voronoi cells are uncorrelated. This is given by twice of the orientationally-averaged radius of the Voronoi cells, i.e., $2r_{eff}$. r_{eff} is given by the average distance from the center to a face of the cell. For the materials under discussion, the Voronoi cells are nearly equiaxed. For a rhombic dodecahedron, which is a suitable representation of these cells, r_{eff} is

related to the cell volume v by standard geometry to be $v_{Vor} = 4\sqrt{2}\, r_{eff}^3$. Hence r_{eff} can be obtained directly from v_{ru}. The results of this analysis is given in Supplementary Table 2.

The rhombic dodecahedron is a suitable representation because maximally random jammed (MRJ) packing of monodispersed spheres of radius r produces twelve nearest neighbors at a distance of just slightly larger than $2r$ and two more distant neighbors at *ca*. $3.4r$ within first adjoining Voronoi layer. However latter two neighbors are really closer to the second shell than the first shell, and hence can be discounted for our purpose of only considering nearest neighbors. The repeat unit shape of the present polymers are not spherical, but closer to prolate, with repeat unit dimension *ca*. 15.7 Å projected along polymer chain, 13.5 Å across width of the triarylamine unit, and 4.5 Å across thickness of the triarylamine unit, giving an oblate aspect ratio of *ca*. 3.2. Such objects are known to be able to give an MRJ state with a packing density ($f \approx 0.66$) similar to those of spheres (≈ 0.64). Although the Voronoi neighbor statistics of these objects do not appear to have been analyzed yet, we anticipate they are closely related to those of spheres. For spheres in the MRJ state, $v_{Vor} = \frac{4}{3}\pi\, r^3 f^{-1}$. For $f = 0.64$, this gives $r_{eff} \approx 1.05\, r$, which is the mean of the pair-correlation function for nearest-neighbor spheres.

The $\langle d_{hh} \rangle$ values turn out to be strongly dependent on the counter-anion size, but only weakly on the pendant ring-substitution studied: 12 Å for the fully-doped polymers with SbF_6^- as counter-anion, and 14 Å for the fully-doped polymers with $BArF^-$ as counter-anion.

The required mean value of nearest-neighbor hole…counter-anion distances $\langle d_{ha} \rangle$ was estimated by molecular modeling of a geometry-optimized triphenylaminium ion at the PM3 level (planar: CN, 1.43 Å; CC, 1.41 Å, 1.39 Å, 1.40 Å; ∠CNC, 120°; phenyl tilt angle *vs* mean plane, 35°) in contact with the desired anion using the MM2 force field or PM3, which are sufficiently accurate for the required precision in the estimation. The results are shown in Supplementary Table 3. Because parameters for the boron atom in BArF are not available in MM2, we used the isoelectronic $C(m,m\text{-}C_6H_3(CF_3)_2)_4$ molecule. The difference between the covalent radii of carbon (0.77 Å) and boron (0.82 Å) atoms is negligible at the precision level of the estimation. Results are shown in Supplementary Table 3.

The closest approach of the SbF_6^- ion to the triphenylaminium nitrogen in the polar direction is 4.6 Å. This yields a realistic van der Waals (vdW) radius of the nitrogen atom of 1.5 Å for a SbF_6^- vdW radius of 3.1 Å.

The closest approach on the molecular equatorial plane is 6.1 Å. The $BArF^-$ ion is much larger. The closest approach in the polar direction is 7.0 Å, and on the molecular plane is 7.2 Å.

Supplementary Note 3

Estimation of the orbital energy shift at a doped hole site due to coulomb potential of counter-ions and other holes

We explain here how we evaluated: (i) through DFT calculations the orbital energy shift at a doped hole site (nitrogen site) in the gas-phase due to the coulomb potential of its nearest counter-anion, and (ii) through classical electrostatic calculations the correction for the presence of other (more distant) counter-anions and holes.

Methodology. Supplementary Note 2 shows that for the hole-doped TAF systems under discussion, the mean distance between nearest-neighbor holes $\langle d_{hh} \rangle$ is larger than the mean distance between an adjacent hole…counter-anion pair $\langle d_{ha} \rangle$ by a factor of two or more even at full doping. Therefore a counter-anion is typically closer to one hole than others, and its hole coordination number is smaller than two, tending towards one. We wish to evaluate the relative shift of the relevant orbital energies of the doped TAF polymers with different counter-anions in solid-state. Direct calculation remains an open problem. Periodic boundary conditions cannot be applied to small cell of an amorphous material. Our approach is to compute the effects of the nearest counter-anion by DFT theory and then correcting for the longer-range interactions with other holes and counter-anions in the classical point-charge approximation. This approach has the further advantage of illuminating the magnitudes and hence importance of various effects.

In the limit that the hole coordination number is one, the energy of a relevant molecular orbital in the repeat unit of the fully hole-doped TAF polymer is given by:

$$u_{MO} = u_{MO,i} + \sum_j u_{MO,j} + u_{MO,pol}, \qquad \text{Supplementary Equation 9}$$

where u_i is the orbital energy that takes into account the presence of a hole in the repeat unit and its associated counter-anion **i** at a specified distance d_{ha}, in the gas phase; $\sum_j u_{MO,j}$ is the sum of coulomb

interactions with other more distant charges **j**, both holes and counter-anions; and $u_{MO,pol}$ is the total polarization energy.

Since the ⟨d_{ha}⟩ of interest is smaller than the spatial extent of the relevant molecular orbitals in the repeat unit (5−7 Å *vs* 10 Å), the $u_{MO,i}$ term needs to be treated self-consistently by quantum chemical calculations taking into account the spatial extent of the orbital, and possible perturbation of the molecular geometry and wavefunction. The relevant orbitals are the empty SOMO' and HOMO of the fully hole-doped state, as explained in Supplementary Note 1. Both these wavefunctions are symmetrically disposed about the nitrogen site and thus behave identically as the singly-occupied SOMO with respect to an approaching anion up to closest realistic approach of 5 Å or so. We thus performed gas-phase energy calculations on the SOMO of a fully hole-doped repeat unit of a model mTFF polymer as a function of the inverse distance to its counter-anion. The repeat unit was modeled by the central portion of a triple-positively charged mTFF trimer with three counter-anions (SbF_6^- or $BArF^-$) positioned directly over the nitrogen atoms symmetrically at various distances perpendicular to the local triphenylaminium plane. The geometric structure of the trimer was optimized at PM6 level. These calculations were performed at the DFT/ CAM-B3LYP/ 3-211G(*dp*) level. $u_{MO,i}$ was obtained by subtracting the coulomb interactions of the central nitrogen site with each of the two terminal hole−counter-anion pairs in the point-charge approximation:

$$U_{MO,i} = E_{MO} - 2\,u_{hh'} - 2\,u_{ha'},$$ Supplementary Equation 10

where E_{MO} is the orbital energy; $u_{hh'} = \frac{e^2}{4\pi\varepsilon_o r_{hh'}}$ is the coulomb interaction with each of the two terminal holes (h'), where $r_{hh'}$ is the distance from the center nitrogen atom to either of the terminal nitrogen atoms; and $u_{ha''} = -\frac{e^2}{4\pi\varepsilon_o r_{ha'}}$ is the coulomb interaction with each of the two terminal counter-anions (a'), where $r_{ha'}$ is the distance from the center nitrogen atom to either of the terminal counter-anions. By removing these coulomb contributions, u_i retains the (extended) coulomb interaction with its associated counter-anion, and any quantum mechanical effects due to orbital overlap with adjacent nitrogen sites.

The u_i energy for the hole…counter-anion repeat unit was thus obtained as a function of d_{ha}, as shown in Supplementary Figure 4. This plot of orbital energy *vs* inverse distance for the hole−counter-anion pair of a

repeat unit gives the correct limiting slope behavior at infinite d_{ha} separation ($\frac{e^2}{4\pi\varepsilon_0}$ = 14.4 eV Å) where the point-charge approximation holds. Significant deviation from this point-charge approximation emerges for $d_{ha} \lesssim 15$ Å due to the extended size of the orbital wavefunction, e.g., for the SOMO, this is −2.0% (−0.20 eV) at d_{ha} = 10 Å. The modification of the hole density distribution itself is rather weak, even at the closest realistic approach. For example, when the counter-anion is 5 Å from the nitrogen atom, the integrated hole density on the adjacent aryl rings increases by only 4%, while that on the pendant ring decreases by 2% and on the fluorene rings decreases 6%.

Since other holes and counter-anions further away does not exhibit any significant electron exchange interaction with the holes, the $\sum_j U_{el,j}$ term can be modeled as purely coulombic in the point-charge first-order approximation. This is reminiscent of the Madelung potential that is well known in ionic solids, but whose role in determining the workfunction of doped organic semiconductors has only recently been pointed out from a study of spectator ion effects. In the absence of long-range order, the magnitude of this term is strongly dominated by the local ion structure. The disordered ion structure beyond a certain short distance does not contribute to the coulomb potential anymore because of complete smear-out of the radial distribution functions.

The local ion structure in the doped TAF polymers cannot be readily measured because of the amorphous nature of these materials. However because of strong coulomb interactions, this structure can be expected to comprise ion clusters, or aggregates of hole…counter-anion pairs, such as charged quartets (two hole…anion pairs), sextets, octets, and higher multiplets, akin to those in ionic liquids with highly asymmetrical ion sizes.

For such an ion cluster, we define the Madelung factor as the ratio of the total electrostatic stabilization energy per cation in the cluster ($u_{el,cat}$) to the electrostatic stabilization energy of an isolated cation−anion pair at closest approach ($u_{el,pair}$): $M = u_{el,cat} / u_{el,pair}$. We considered 1:1 neutral ion clusters comprising two (i.e., ion quartet) to 512 ion pairs in various high-symmetry cation sub-lattices. The sub-lattice parameter was systematically dilated to simulate geometric constraints on the holes. The positions of the anions were then

optimized to the cation sub-lattice. Na^+ and Cl^- ions were employed as models to impose realistic "hard" sphere size-exclusion potentials. The simulations were conveniently performed using molecular mechanics (MM2) force fields. The Madelung factor obtained was then plotted against the d_1/d_2 ratio, where d_1 is the nearest-neighbor cation...cation distance, and d_2 is the nearest-neighbor cation...anion distance. The results are shown in Supplementary Figure 5. The M values obtained here for these constraint 1:1 ion clusters are considerably smaller than those for NaCl and CsCl lattices (1.75−1.76) because of finite cluster effects and huge ion-size asymmetry that prevents close ion packing. Crucially the values of M for $d_1 / d_2 \gtrsim 1.8$ are largely independent of cluster morphology for sizes larger than a quartet. The limit of M for large d_1 / d_2 is unity, as expected. This means that for the hole-doped triarylaminium copolymer systems, M is determined primarily by the d_{hh} to d_{ha} ratio, rather than the unknown morphology of the hole…counter-anion cluster.

The $\sum_j U_{el,j}$ term is thus given by: $-\frac{e^2}{4\pi\varepsilon_0\varepsilon_r\langle d_{ha}\rangle}(M\text{-}1)$, where ($M$−1) gives the contributions by all other holes and their associated counter-anions, and ε_r is the static dielectric constant.

The evaluation of u_i and $\sum_j u_{el,j}$ for the expected $\langle d_{hh}\rangle$ and $\langle d_{ha}\rangle$ parameters for the fully hole-doped triarylaminium polymers counter-balanced by SbF_6^- or $BArF^-$ are collected in Supplementary Table 4. There is practically no dependence on the pendant-ring substitution studied.

Assuming that the true local structure of the hole…counter-anion pair is an average of the polar and equatorial closest approaches, the average $u_i + \sum_j u_{el,j}$ for SbF_6^- as counter-anion is −9.08 eV and for $BArF^-$ as counter-anion is −9.51 eV. Since u_{pol} should be practically constant, the orbital energies for the $BArF^-$ counter-anion can be expected to be *ca*. 0.4 eV deeper than for the SbF_6^- counter-anion. The shift is accounted primarily by loss of coulomb stabilization of the hole by the larger size of the nearest-neighbor $BArF^-$ counter-anion, with little differences in the contributions from holes and counter-anions further away. The agreement with experiment is remarkable for this simple zero-free-parameter model.

Supplementary Note 4

Estimation of polymer repeat unit volumes (r_{ru})

mTFF has a repeat unit given by $C_{47}F_3H_{52}N$ which corresponds to a molecular weight of 688 g mol^{-1}. The repeat unit volume v_{ru} can be estimated by additive group molar volume contributions as shown in Supplementary Table 5 to be 646 cm^3 mol^{-1}. This gives a density of 688 g mol^{-1} / 646 cm^3 mol^{-1} = 1.06 g cm^{-3}. Measurement gives 1.03 ± 0.01 g cm^{-3} (flotation method). This validates the group contribution method for density estimation in this family of polymers to better than 3%. Uncertainty in linear dimensions is thus less than 1%. The density corresponds to 9.35 x 10^{20} repeat units per cm^3, and a repeat unit volume of 1.07 x 10^{-21} cm^3.

Similar calculations for other polymers in our study gave the results in Supplementary Table 6.

The SbF_6^- ion is octahedral with an effective vdW radius of 3.1 ± 0.1 Å. The ion is expected to be freely rotating to generate an excluded vdW volume of 0.125 x 10^{-21} cm^3. This value is consistent with a Sb−F bond length is 1.9 Å (DFT calculations performed at B3LYP/ 3-21G) and an vdW radius of F of 1.3 Å.

The BArF$^-$ ion is tetrahedral. This ion may not be freely rotating. Therefore its vdW volume may be estimated from group molar volume contributions. The result is 479 cm^3 mol^{-1} (Supplementary Table 7), which corresponds to a molecular volume of 0.80 x 10^{-21} cm^3. By equating this to the volume of a sphere, the effective vdW radius was found to be 5.8 Å. This is consistent with MM2 estimates of the size of this ion. The closest polar approach distance between BArF$^-$ and triphenylaminium is 7.0 Å, which indicates an effective radius of 5.5 Å. This is smaller than the sphere radius because the excluded volume is disposed only in the tetrahedral directions.

The group contribution parameters have been validated using tetraphenylmethane as a molecular model. Its vdW volume from group-contribution additivity is 243 cm^3 mol^{-1} (Supplementary Table 8), which for a molecular weight of 244.3 g mol^{-1} gives a density of 1.00 g cm^{-3}. The experimental value is 1.01 g cm^{-3}.
